# Childhood obesity and sleep: relatives, partners, or both?—a critical perspective on the evidence

**DOI:** 10.1111/j.1749-6632.2012.06723.x

**Published:** 2012-08-01

**Authors:** David Gozal, Leila Kheirandish-Gozal

**Affiliations:** Department of Pediatrics and Comer Children's Hospital, Pritzker School of Medicine, The University of ChicagoChicago, Illinois

**Keywords:** sleep, obesity, children, sleep apnea

## Abstract

In modern life, children are unlikely to obtain sufficient or regular sleep and waking schedules. Inadequate sleep affects the regulation of homeostatic and hormonal systems underlying somatic growth, maturation, and bioenergetics. Therefore, assessments of the obesogenic lifestyle, including as dietary and physical activity, need to be coupled with accurate evaluation of sleep quality and quantity, and coexistence of sleep apnea. Inclusion of sleep as an integral component of research studies on childhood obesity should be done as part of the study planning process. Although parents and health professionals have quantified normal patterns of activities in children, sleep has been almost completely overlooked. As sleep duration in children appears to have declined, reciprocal obesity rates have increased. Also, increases in pediatric obesity rates have markedly increased the risk of obstructive sleep apnea syndrome (OSAS) in children. Obesity and OSAS share common pathways underlying end-organ morbidity, potentially leading to reciprocal amplificatory effects. The relative paucity of data on the topics covered in the perspective below should serve as a major incentive toward future research on these critically important concepts.

## Sleep trends in children and potential associations with obesity

Although sleep assessments of children have largely relied on parental reports,^1^–^7^ it has become apparent that children are highly unlikely to obtain sufficient sleep on a stable and regular schedule. Polls by the National Sleep Foundation^1^,^2^ show that parents routinely overestimate their children's sleep duration, and that in fact children sleep much less than what is deemed appropriate for their age. At the society level, and likely across all pediatric ages, children sleep less than what they did one century ago.^8^ Despite the compelling evidence supporting a vital role for healthy sleep in brain maturation, somatic growth, information processing, memory consolidation, learning, and other important neurobehavioral functions, parents and professionals instead focus their attention on children's accomplishments (e.g., first steps, first words, school grades, extracurricular activity performances), and often treat sleep as a tradable commodity.

Clearly, disturbed sleep patterns can lead to multidimensional adverse effects. For example, 43% of school-aged children and 57% of adolescents have a TV in their bedroom;^1^,^2^,^9^ and as many as 42% have mobile phones in their bedroom and many other electronic devices, such as computers, video games, and other electronic gadgetry are also frequently present in bedrooms.^9^,^10^ These devices account for multiple intrusion patterns that can curtail or disrupt sleep in children and may result in the development of decreased opportunities for sleep in children over time—put differently, it could be said that modern life has “polluted” the opportunity to sleep because of a variety of intrusions, which may further lead to reduced sleep duration or sleep disruption.

In the last 20 years, a large number of studies have been published on sleep duration in children, and most have aimed to evaluate the potentially adverse impact of poor sleep on health outcomes. Although these studies are heterogeneous in their methodology and scope, they all point to the critical need for urgent large-scale representative and longitudinal studies on objective sleep–wake patterns in children, particularly to explore the impact of sleep on health and body weight in a valid ecological and contextual setting. For example, the family domain, important with respect to sleep–wake patterns,^11^,^12^ influences food and health-related behaviors in the developing child. Not surprisingly, the presence of parental beliefs about being overweight and nutrition can have significant impact on the risk of childhood obesity.^13^,^14^ Because daily activities and sleep patterns evolve in a circadian continuum, parenting feeding styles^15^ may interact with sleep. For instance, when U.S. preschoolers were exposed to three household routines, namely, evening family meal for more than 5 nights per week, sleeping ≥10.5 h/night on weekdays, and ≤2 h/day television, video, or other screen-viewing behavior, obesity prevalence decreased by 40% compared with when no such simple routines were present.^16^

**Figure 1:**
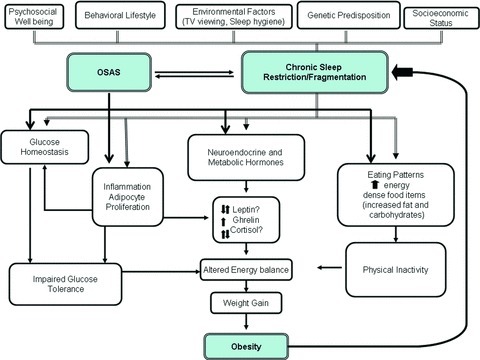
Putative interactions between reduced or fragmented sleep, sleep apnea, and obesity in children.

The estimated dose–response relationship between sleep and obesity is highly variable,^17^ with pooled odd ratios ranging from 1.15 to 11.0, which likely represents heterogeneities in methodologies and the diversity of contributing factors (Fig. 1).^18^ In a recent study in which sleep and weight were carefully monitored using objective assessments, we found that irregular and shorter sleep is a significant risk factor for the occurrence of weight problems in children, and that a nonlinear trend between sleep and weight was present.^19^ We further found that obese children are less likely to catch-up in their sleep “debt” during weekends, and that the combination of shorter sleep duration and more variable sleep patterns was associated not only with increased weight risk but also with adverse metabolic outcomes (i.e., insulin resistance, elevated serum lipids, and increased high-sensitivity C reactive protein levels).

The biological correlates of the association between sleep patterns and body mass index (BMI) have been partially elucidated and would support the assumption that either inadequate amounts of sleep or disrupted sleep architecture lead to alterations in some of the neuropeptides that regulate appetite, such as increased levels of ghrelin, reduced levels of leptin, and reduced central biological activity of orexin, with the anticipated cumulative effect of promoting increased food intake (Fig. 1).^20^,^21^

The association between short sleep duration and increased risk for obesity is, as mentioned, somewhat conflictive, whereby discrepancies also emerge in relation to age. For example, although a significant contribution of sleep duration to obesity risk is present in adults, the associations are, as indicated above, highly variable and could reflect a multitude of possible confounders that may be operational in early life.^22^–^28^ Furthermore, in an elegant and important cross-sectional and longitudinal study, Chaput and colleagues reported that only those adults manifesting short sleep duration, highly disinhibited eating behaviors, and/or low dietary calcium intake had significantly higher BMI compared with corresponding controls. Indeed, over the six-year follow-up period, such high-risk adults were significantly more likely to gain weight and develop obesity.^18^

Notwithstanding these considerations, a recent meta-analysis of the literature appears to support the conclusion that the strength of the association between sleep duration and obesity is actually stronger in children and adolescents, and that it declines over time.^29^ Furthermore, some degree of predisposition for the existence of such association has been proposed in light of the findings that sleep-associated changes in BMI appear to be primarily occurring in children whose BMI was already elevated.^30^

We should emphasize that the majority of these studies have, as mentioned above, relied on subjective, parentally reported estimates of sleep duration,^31^–^33^ and that the effects of variability of sleep schedules on BMI has not been explored. Also, parental reports generally overestimate sleep duration of children.^32^–^36^ In addition, we are unaware of experimental studies that aim to characterize the effect of sleep manipulations on metabolic homeostasis in children. Indeed, the definition of what constitutes “short sleep” is arbitrary and highly variable across different people and across various studies, further adding to the complexity of the critical assessment of the association between sleep and BMI. A given “sleep duration” over a relatively large range may in fact be considered long and sufficient sleep in one child, although short and insufficient sleep in another.

Regardless of the aforementioned limitations, the association of sleep duration with BMI generally exhibits about a 1.5- to twofold increase in the likelihood of being a short sleeper when obesity is present.^37^–^39^ And although interventional studies aiming to modify sleep patterns in children are clearly needed, they may be fraught with substantial failure rates, particularly considering that both sleep regularity and sleep duration are maintained across long periods of time during childhood; and thus any intervention will likely need to be initiated very early in life if the effect is to be measurable.^39^ Accordingly, identification of particular young children at high risk and prospective interventions aiming to prolong and regularize sleep in these children will provide more definitive evidence regarding the role of sleep in BMI and metabolic regulation. Even if such studies are conducted, it will take a long time before their findings can be incorporated into clinical practice. Accordingly, based on the current, albeit deficient level of knowledge, we feel that it might be worth advocating for implementing educational campaigns aimed at families and health professionals, campaigns that target the promotion of longer and regular sleep habits among toddlers and beyond.

Although not exactly within the scope of this paper, it is worthwhile mentioning that strong evidence supporting the biological plausibility of a strong link between sleep, appetite regulation, and adiposity has been made over the last few years. For example, circadian clocks are integral regulators of cellular metabolism that also modulate both appetite and food intake in both animals and humans.^40^–^42^ Perturbations of the endogenous global or organ-specific circadian cycle, or alterations in the integrity of sleep homeostatic mechanisms, therefore increases the risk of altered energy intake and disposition, which may ultimately lead to increased propensity for developing obesity and metabolic disturbances.

## Obesity and obstructive sleep apnea syndrome

Since its initial description in 1976, obesity and obstructive sleep apnea syndrome (OSAS) has become widely recognized as a highly prevalent condition in children.^43^ OSAS has now been recognized as leading to a spectrum of potentially serious morbidities affecting the central nervous, cardiovascular, and metabolic systems, and studies exploring the potential mechanisms leading to end-organ dysfunction in the context of OSAS indicate that both oxidative stress and inflammatory processes are operational. The interplay of these processes with disease severity, environmentally related modifiers, and individual genetic susceptibility is clearly emerging as the optimal model accounting for phenotypic variance.^44^–^48^ In parallel, the last two decades has witnessed a shift from the classic presentation of children with OSAS (i.e., adenotonsillar hypertrophy and failure to thrive) to a majority of children being overweight or obese, even though adenotonsillar hypertrophy continues to play a role in the latter group.^49^

OSAS in children is characterized by recurrent events of partial or complete upper airway obstruction during sleep, resulting in disruption of normal gas exchange (intermittent hypoxia and hypercapnia) and of sleep through multiple arousals, leading to sleep fragmentation. Although enlarged tonsils and adenoids in the upper airway clearly play a role,^50^ it has now become evident that the interplay between alterations in structural and anatomical characteristics, upper airway mucosal properties and inflammation, and protective reflexes and neuromotor abnormalities of the upper airway are the major determinants of whether upper airway obstruction will develop during sleep, as well as its severity and frequency. The clinical spectrum of obstructive sleep-disordered breathing includes frank OSAS of varying severity, upper airway resistance syndrome (traditionally associated with low-frequency obstructive apneic events and globally preserved normal oxygenation patterns but increased respiratory-related sleep fragmentation), and, at the low end of the severity spectrum, a condition that has been termed either primary or habitual snoring (i.e., habitual snoring in the absence of apneas, gas exchange abnormalities, and/or disruption of sleep architecture). The prevalence of OSAS in children is currently estimated to be ∼3% among 2- to 8-year-old children.^51^ However, habitual snoring during sleep—the hallmark of increased upper airway resistance—is much more prevalent.^52^,^53^

More recently, obesity has been shown to markedly increase the risk of OSAS,^54^–^60^ whereby upper airway narrowing may be the consequence of fatty infiltration of upper airway structures and the tongue, whereas subcutaneous fat deposits in the anterior neck region and other cervical structures also exert force vectors promoting increased pharyngeal collapsibility.^61^,^62^ Increased adipose tissue mass in the abdominal wall and cavity, as well as in the thorax, increases the global respiratory load and reduces intrathoracic diaphragm excursion, particularly during the supine position, leading to decreased lung volumes and oxygen reserve, and increased work of breathing during sleep. Furthermore, obesity can be accompanied by poor quality sleep, which, in turn, can perturb arousal mechanisms and, therefore, delay upper airway opening, thus exacerbating the duration of apnea.^63^

As mentioned, the presence of OSAS may promote leptin resistance and enhance ghrelin levels, both of which can perpetuate the tendency for obesogenic behaviors.^64^,^65^ The unidirectional drivers of obesity—increasing the probability of OSAS—appear to be functional in the reverse direction as well. In other words, OSAS may either promote or exaggerate the tendency for obesity, or its consequences (Fig. 1). For example, OSAS is associated with daytime sleepiness, and sleepiness is exacerbated when obesity is concurrently present.^66^,^67^ In addition, sleepiness will reduce the likelihood of engaging in physical activity and enhance obesogenic eating behaviors that favor calorie-dense foods, particularly in those children at risk for obesity.^65^ Finally, OSAS is a chronic low-grade inflammatory disease that interacts with and potentiates obesity-induced inflammatory processes.^68^–^71^

We should again emphasize that despite the rather compelling evidence supporting a bidirectional interaction between OSAS and obesity, there are no well-controlled interventional trials that have assessed whether effective treatment of OSAS will improve obesity risk and obesity-related outcomes, and conversely, whether improvements in obesity are associated with improvements in OSAS severity.^59^,^72^,^73^

## Conclusions

In summary, sleep and body weight appear to share a constellation of contributing factors that originate in the spectrum encompassed by child, family, and society, and in which food intake patterns, physical activity, and sleep habits emerge as integral contributors to obesity risk. Unfortunately, the relative contribution of poor sleep remains virtually unexplored—a fact that should prompt renewed efforts to elucidate the mechanisms underlying metabolic dysregulation in the context of short sleep, irregular sleep, or disrupted sleep. The nature and scope of these interactions and the potential mechanisms underlying such putative associations will need to be explored much more thoroughly and carefully in the near future, to enable formulation of realistic and cogent public intervention programs aimed at reducing the unacceptably high rates of obesity in children. We have also discussed the putative presence of reciprocal contributions of OSAS and obesity that perpetuate and enhance each other.

There is little doubt that more precise understanding of the effects of sleep disruption and tissue hypoxia on the phenotypic expression of these diseases is warranted. The assessment and identification of unique genomic, proteomic, and metabolomic pathways underlying the antecedents and consequences of these highly prevalent pediatric diseases appears necessary and before implementing sound and valid treatment strategies.

## References

[1] National Sleep Foundation (2004). Sleep in America poll–children and sleep. http://www.sleepfoundation.org/article/sleep-america-polls/2004-children-and-sleep.

[2] National Sleep Foundation (2006). Sleep in America poll—teens and sleep. http://www.sleepfoundation.org/article/sleep-america-polls/2006-teens-and-sleep.

[3] Knutson KL, Lauderdale DS (2009). Sociodemographic and behavioral predictors of bed time and wake time among US adolescents aged 15 to 17 years. J. Pediatr.

[4] Dollman J, Ridley K, Olds T, Lowe E (2007). Trends in the duration of school-day sleep among 10- to 15-year-old South Australians between 1985 and 2004. Acta Paediatr.

[5] Sadeh A, Raviv A, Gruber R (2000). Sleep patterns and sleep disruptions in school-age children. Dev. Psychol.

[6] Yang CK, Kim JK, Patel SR, Lee JH (2005). Age-related changes in sleep/wake patterns among Korean teenagers. Pediatrics.

[7] Warner S, Murray G, Meyer D (2008). Holiday and school-term sleep patterns of Australian adolescents. J. Adolesc.

[8] Matricciani LA, Olds TS, Blunden S (2012). Never enough sleep: a brief history of sleep recommendations for children. Pediatrics.

[9] Spruyt K, Slaapproblemen. bij. kinderen (2007). Basisgids Voor Ouders en Hulpverleners (Sleep problems in children. A clinical guide for parents and health care providers).

[10] Li S, Jin X, Wu S (2007). The impact of media use on sleep patterns and sleep disorders among school-aged children in China. Sleep.

[11] El-Sheikh M, Buckhalt JA, Keller PS (2007). Child emotional insecurity and academic achievement: the role of sleep disruptions. J. Fam. Psychol.

[12] El-Sheikh M, Buckhalt JA, Mark Cummings E, Keller P (2007). Sleep disruptions and emotional insecurity are pathways of risk for children. J. Child. Psychol. Psychiatry.

[13] Cole TJ (2006). The international growth standard for preadolescent and adolescent children: statistical considerations. Food. Nutr. Bull.

[14] Baughcum AE, Chamberlin LA, Deeks CM (2000). Maternal perceptions of overweight preschool children. Pediatrics.

[15] Chassin L, Presson CC, Rose J (2005). Parenting style and smoking-specific parenting practices as predictors of adolescent smoking onset. J. Pediatr. Psychol.

[16] Anderson SE, Whitaker RC (2010). Household routines and obesity in US preschool aged children. Pediatrics.

[17] Chen XBM, Wang Y (2008). Is sleep duration associated with childhood obesity? A systematic review and meta-analysis. Obesity (Silver Spring).

[18] Cappuccio FP, Taggart FM, Kandala NB (2008). Meta-analysis of short sleep duration and obesity in children and adults. Sleep.

[19] Spruyt K, Molfese DL, Gozal D (2011). Sleep duration, sleep regularity, body weight, and metabolic homeostasis in school-aged children. Pediatrics.

[20] Zheng H, Berthoud H-R (2008). Neural systems controlling the drive to eat: mind versus metabolism. Physiology.

[21] Mavanji V, Teske JA, Billington CJ, Kotz CM (2010). Elevated sleep quality and orexin receptor mRNA in obesity-resistant rats. Int. J. Obes. (Lond.).

[22] Hairston KG, Bryer-Ash M, Norris JM (2010). Sleep duration and five-year abdominal fat accumulation in a minority cohort: the IRAS family study. Sleep.

[23] Grandner MA, Patel NP, Gehrman PR (2010). Problems associated with short sleep: bridging the gap between laboratory and epidemiological studies. Sleep Med. Rev.

[24] Monasta L, Batty GD, Cattaneo A (2010). Early-life determinants of overweight and obesity: a review of systematic reviews. Obes. Rev.

[25] Nishiura C, Noguchi J, Hashimoto H (2010). Dietary patterns only partially explain the effect of short sleep duration on the incidence of obesity. Sleep.

[26] Taveras EM, Rifas-Shiman SL, Rich-Edwards JW (2011). Association of maternal short sleep duration with adiposity and cardiometabolic status at 3 years postpartum. Obesity (Silver Spring).

[27] Buxton OM, Marcelli E (2010). Short and long sleep are positively associated with obesity, diabetes, hypertension, and cardiovascular disease among adults in the United States. Soc. Sci. Med.

[28] Taveras EM, Rifas-Shiman SL, Rich-Edwards JW, Mantzoros CS (2011). Association of maternal short sleep duration with adiposity and cardiometabolic status at 3 years postpartum. Metabolism.

[29] Nielsen LS, Danielsen KV, Sørensen TI (2011). Short sleep duration as a possible cause of obesity: critical analysis of the epidemiological evidence. Obes. Rev.

[30] Bayer O, Rosario AS, Wabitsch M, von Kries R (2009). Sleep duration and obesity in children: is the association dependent on age and choice of the outcome parameter?. Sleep.

[31] Nixon GM, Thompson JMD, Han DY (2008). Short sleep duration in middle childhood: risk factors and consequences. Sleep.

[32] Marshall NS, Glozier N, Grunstein RR (2008). Is sleep duration related to obesity? A critical review of the epidemiological evidence. Sleep Med. Rev.

[33] Marshall NS, Glozier N, Grunstein RR (2008). Reply to Taheri and Thomas: is sleep duration associated with obesity-U cannot be serious. Sleep Med. Rev.

[34] Patel SR, Hu FB (2008). Short sleep duration and weight gain: a systematic review. Obesity.

[35] Gupta NK, Mueller WH, Chan W, Meininger JC (2002). Is obesity associated with poor sleep quality in adolescents?. Am. J. Hum. Biol.

[36] Dayyat EA, Spruyt K, Molfese DL, Gozal D (2011). Sleep estimates in children: parental versus actigraphic assessments. Nat. Sci. Sleep.

[37] Padez C, Mourao I, Moreira P, Rosado V (2009). Long sleep duration and childhood overweight/obesity and body fat. Am. J. Hum. Biol.

[38] Taveras EM, Rifas-Shiman SL, Oken E (2008). Short sleep duration in infancy and risk of childhood overweight. Arch. Pediatr. Adolesc. Med.

[39] Touchette E, Petit D, Tremblay RE (2008). Associations between sleep duration patterns and overweight/obesity at age 6. Sleep.

[40] Huang W, Ramsey KM, Marcheva B, Bass J (2011). Circadian rhythms, sleep, and metabolism. J. Clin. Invest.

[41] Leproult R, Van Cauter E (2010). Role of sleep and sleep loss in hormonal release and metabolism. Endocr. Dev.

[42] Hanlon EC, Van Cauter E (2011). Quantification of sleep behavior and of its impact on the cross-talk between the brain and peripheral metabolism. Proc. Natl. Acad. Sci. USA.

[43] Guilleminault C, Eldridge FL, Simmons FB, Dement WC (1976). Sleep apnea in eight children. Pediatrics.

[44] Capdevila OS, Kheirandish-Gozal L, Dayyat E, Gozal D (2008). Pediatric obstructive sleep apnea: complications, management, and long-term outcomes. Proc. Am. Thorac. Soc.

[45] Gozal D, Kheirandish-Gozal L (2008). The multiple challenges of obstructive sleep apnea in children: morbidity and treatment. Curr. Opin. Pediatr.

[46] Kheirandish-Gozal L, Bhattacharjee R, Gozal D (2010). Autonomic alterations and endothelial dysfunction in pediatric obstructive sleep apnea. Sleep Med.

[47] Kim J, Hakim F, Kheirandish-Gozal L, Gozal D (2011). Inflammatory pathways in children with insufficient or disordered sleep. Respir. Physiol. Neurobiol.

[48] Bhattacharjee R, Kim J, Kheirandish-Gozal L, Gozal D (2011). Obesity and obstructive sleep apnea syndrome in children: a tale of inflammatory cascades. Pediatr. Pulmonol.

[49] Dayyat E, Kheirandish-Gozal L, Gozal D (2007). Childhood obstructive sleep apnea: one or two distinct disease entities?. Sleep Med. Clin.

[50] Katz ES, D’Ambrosio CM (2008). Pathophysiology of pediatric obstructive sleep apnea. Proc. Am. Thorac. Soc.

[51] Lumeng JC, Chervin RD (2008). Epidemiology of pediatric obstructive sleep apnea. Proc. Am. Thorac. Soc.

[52] Ferreira AM, Clemente V, Gozal D (2000). Snoring in Portuguese primary school children. Pediatrics.

[53] O’Brien LM, Holbrook CR, Mervis CB (2003). Sleep and neurobehavioral characteristics of 5- to 7-year-old children with parentally reported symptoms of attention-deficit/hyperactivity disorder. Pediatrics.

[54] Arens R, Muzumdar H (2010). Childhood obesity and obstructive sleep apnea syndrome. J. Appl. Physiol.

[55] Redline S, Tishler PV, Schluchter M (1999). Risk factors for sleep-disordered breathing in children. Associations with obesity, race, and respiratory problems. Am. J. Respir. Crit. Care. Med.

[56] Wing YK, Hui SH, Pak WM (2003). A controlled study of sleep related disordered breathing in obese children. Arch. Dis. Child.

[57] Kalra M, Inge T, Garcia V (2005). Obstructive sleep apnea in extremely overweight adolescents undergoing bariatric surgery. Obes. Res.

[58] Bixler EO, Vgontzas AN, Lin HM (2009). Sleep disordered breathing in children in a general population sample: prevalence and risk factors. Sleep.

[59] Verhulst SL, Franckx H, Van Gaal L (2009). The effect of weight loss on sleep-disordered breathing in obese teenagers. Obesity (Silver Spring).

[60] Dayyat E, Kheirandish-Gozal L, Sans Capdevila O (2009). Obstructive sleep apnea in children: relative contributions of body mass index and adenotonsillar hypertrophy. Chest.

[61] Horner RL, Mohiaddin RH, Lowell DG (1989). Sites and sizes of fat deposits around the pharynx in obese patients with obstructive sleep apnoea and weight matched controls. Eur. Respir. J.

[62] White DP, Lombard RM, Cadieux RJ, Zwillich CW (1985). Pharyngeal resistance in normal humans: influence of gender, age, and obesity. J. Appl. Physiol.

[63] Beebe DW, Lewin D, Zeller M (2007). Sleep in overweight adolescents: shorter sleep, poorer sleep quality, sleepiness, and sleep-disordered breathing. J. Pediatr. Psychol.

[64] Tauman R, Serpero LD, Capdevila OS (2007). Adipokines in children with sleep disordered breathing. Sleep.

[65] Spruyt K, Sans Capdevila O, Serpero LD (2010). Dietary and physical activity patterns in children with obstructive sleep apnea. J. Pediatr.

[66] Tauman R, O’Brien LM, Holbrook CR, Gozal D (2004). Sleep pressure score: a new index of sleep disruption in snoring children. Sleep.

[67] Gozal D, Kheirandish-Gozal L (2009). Obesity and excessive daytime sleepiness in prepubertal children with obstructive sleep apnea. Pediatrics.

[68] Gozal D, Serpero LD, Sans Capdevila O, Kheirandish-Gozal L (2008). Systemic inflammation in non-obese children with obstructive sleep apnea. Sleep Med.

[69] Kim J, Bhattacharjee R, Snow AB (2010). Myeloid-related protein 8/14 levels in children with obstructive sleep apnoea. Eur. Respir. J.

[70] Spruyt K, Gozal D (2012). A mediation model linking body weight, cognition, and sleep-disordered breathing. Am. J. Respir. Crit. Care. Med.

[71] Bhattacharjee R, Kim J, Alotaibi WH (2012). Endothelial dysfunction in children without hypertension: potential contributions of obesity and obstructive sleep apnea. Chest.

[72] Gozal D, Capdevila OS, Kheirandish-Gozal L (2008). Metabolic alterations and systemic inflammation in obstructive sleep apnea among nonobese and obese prepubertal children. Am. J. Respir. Crit. Care. Med.

[73] Bhattacharjee R, Kheirandish-Gozal L, Spruyt K (2010). Adenotonsillectomy outcomes in treatment of obstructive sleep apnea in children: a multicenter retrospective study. Am. J. Respir. Crit. Care. Med.

